# American Dental Association

**Published:** 1884-09

**Authors:** Chas. Mayr


### AMERICAN DENTAL ASSOCIATION.

### TWENTY-FOURTH ANNUAL MEETING, AT SARATOGA.

REPORTED FOR THE INDEPENDENT PRACTITIONER BY PROF. CHAS. MAYR.

The meeting opened at 11 o'clock Tuesday, August 5, President E. T. Darby in the chair.

Prayer was offered by Rev. Dr. Cary of Saratoga.

After the calling of the roll it was voted to dispense with the reading of the minutes of the last meeting. The chairman of the committee on arrangements reported the programme as follows: Daily sessions from 9.30 A. M. to 2 P. M., and from 8 P. M. to adjournment.

It was on motion voted to have a clinical session Wednesday afternoon.

The Secretary, Dr. Cushing, reported amendments to the constitution, and asked unanimous consent for their immediate adoption. Section 2 of Article 6 to be so amended as to make it the duty of every permanent member to inform the Recording Secretary, before the close of the *second day* of the meeting, of the section which he desires to join. Other amendments provided for the early organization of the sections, the object of the whole being to secure a more perfect organization of the various sections. The amendments were unanimously adopted.

The President then read his annual address, and upon motion of Dr. McKellops it was referred to a committee of five, consisting of Drs. Brown, Harlan, Taft, Foster and Pierce for special consideration and report.

On motion of Dr. Ambler a committee of three was appointed to draft suitable resolutions relative to the death of Dr. T. L. Buckingham. The President appointed as such committee Drs. Ambler Allen and Taft.

*Dr. C. N. Pierce*—Called attention to the fact that there would be an adjourned session of the meeting of the Faculties of the Dental Colleges during the afternoon.

*Dr. Geo. H. Cushing,* chairman—Read the report of the Committee on Publication.

*Dr. C. D. Cooke*—Called attention to the fact that the names of a number of men were reported as members of sections who were not members of the Association.

*The Secretary*—Announced that he had retained the names of some who had allowed their membership to lapse, but in every case he had reason to believe it was through inadvertence, and not from a desire to withdraw from the Society. Adjourned.

TUESDAY EVENING SESSION.

The meeting was called to order by the President.

It was voted that Wednesday evening's session be devoted to the paper on Histology, by Dr. J. L. Williams, with its stereopticon illustrations.

Section VI., "Pathology, Therapeutics and Materia Medica," was then called, and the report was presented by the chairman, Dr. A. W. Harlan. The subject was Pyorrhœa Alveolaris. The author said that the report was but a continuation of the paper of last year, bringing out details suggested by numerous inquiries. He was gratified to know that his efforts have been so spontaneously recognized, and expressed his acknowledgment. Many in the profession are liable to confound salivary deposits on the lower incisors, or on the palatal or labial surfaces of molars, with pyorrhœa alveolaris. These are only in a slight degree the cause of, or associated with, this affection. In all cases of abundant discharge there may be observed a loss of tissue around the teeth, whereby their symmetrical outline becomes irregular, and this finally leads to extensive destruction of the bone.

It is not a disease of old age, any more than of youth or middle age. I have seen it as early as the ninth year, and several times before the sixteenth year. My experience leads me to think that between the ages of twenty-five and forty it is most frequent. Pulpless teeth do not escape the ravages; users of tobacco are not less liable to attack than those who abstain. In the course of the disease the teeth twist, change position, and protrude. If thoughtlessly extracted, the features may be greatly altered. The fact remains that a vast majority of cases is unaccompanied with salivary calculus. In most cases the gums have a purplish color, corresponding to the shape of the pockets. As regards the treatment, experience has shown the necessity for the use of instruments of extreme delicacy to examine the pockets for sanguinary deposits, etc.; if salivary calculus is found, it should be carefully removed and the case treated with the following preparation:

| | |
|---|---|
| Pinus canadensis, | ½ oz. |
| Aqua rosae, | 1 oz. |
| Eugenol, | 30 drops. |

to be applied twice daily.

To remove the calculus I use Cushing's scalers, necessitating a pushing motion. In addition, two or three properly shaped excavators are necessary to scrape the edge of the alveolus. A fine pointed syringe is needed, the point not too sharp, with the opening near instead of at the point. $H_2 O_2$ (hydrogen peroxide) ought to be injected immediately after removal of deposits or excision of the edges of the processes. I also use iodide of zinc in solution, three to forty-eight grains to one ounce of water, and begin by the injection of three to four drops into each pocket, after the injection of the $H_2 O_2$. I have also used Eugenol and Sanitas oil in place of hydrogen peroxide. I direct my patients to brush the gums with a stiff brush, and discontinue the use of the pinus canadensis after the beginning of the treatment of the pockets. Loose teeth are bound together with binding wire of silver, platinum, or gold. When the suppuration ceases, the injection of hydrogen peroxide is discontinued, and a dentifrice of the following formula employed:

| | |
|---|---|
| Precipitated chalk, | 2 oz. |
| Orris root, | 2 oz. |
| Castile soap, | ½ oz. |
| Powdered borax, | ½ oz. |
| Powdered myrrh, | ½ oz. |

Glycerine and honey, enough to form a paste.

If desired, I add about twenty drops of Eugenol. After the fourth or fifth visit the interval may be longer; once a week, and so gradually increased. As stated before, in all cases of pyorrhœa with pain, Iodoform made into a paste with Oil of Cinnamon and Oil of Eugenol is an excellent local anæsthetic. I have also had good results from injection of a weak solution of Chloride of Alumina, one to two grains to one ounce of water. Iodo-chloride of zinc, a saturated solution of Iodine, Wood Creosote, Iodoform, Eucalyptol, a diluted alcoholic solution of Menthol, etc, have also been used, but I prefer the first mentioned remedies.

The second portion of the report was upon the qualities and character of Eugenol and Oil Sanitas. Eugenol is the essential principle of Oil of Cloves.

It is known among chemists and in the dispensatories as Eugenic acid. It is prepared by decomposing Eugenate of Potassium with Sulphuric acid; it is then rectified. An oil of low specific gravity is obtained. It has the odor of the oil of cloves and the composition $C_{10} H_{12} O_2$. It is not decomposed at ordinary temperature, has no tendency to become thick on exposure to air, or to precipitate a sediment. It may be diluted with water or alcohol. It is a powerful parasiticide. When applied to an exposed pulp the pain is greatly reduced, and soon ceases altogether. If the cavity is previously washed with Biborate of Soda, it is probably the best dressing for slightly inflamed pulps, not excepting the various iodoform pastes. It may be injected into fistulous tracts, and the cavity sealed immediately. I have injected three drops into the interior of an epulis, and in three weeks it has disappeared. As an injection into the root it should supersede all the powerful coagulators; diluted with water, 1 to 1,000, it forms an agreeable dressing, and may be substituted for $H_2 O_2$. With proper precaution it may be injected full strength into an abscess without danger.

Sanitas oil is obtained by the oxidation of oil of turpentine. It is produced from turpentine floating on water, by directing a stream of heated air on its surface. It contains an empyreumatic oil, and a substance probably identical with $H_2 O_2$. It possesses oxidizing power equal to a ten volume solution of $H_2 O_2$, and may be considered as a convenient method of storing it. In it we have a germicide of the first rank. Combined with volatile oils it gives a dressing which is disinfecting, antiseptic, and not poisonous. It has the odor of fresh pines, which may be disguised by oil of Gaultheria, Eugenol, or Eucalyptol, but a large volume is needed. It has been used for injection into the pyorrhœa pockets in the treatment of alveolar abscess, and as an ingredient of mouth washes for the treatment of recent wounds, and as a dressing over exposed pulps. It is soluble in alcohol and ether, and some of the essential oils. Eugenol may be procured from Seward and Merks, while Sanitas is made by the Sanitas Company, in London, England, and is imported by Sargent, Chicago.

(TO BE CONTINUED.)

